# Evaluation of Internal Fit and Marginal Adaptation of Provisional Crowns Fabricated with Three Different Techniques

**DOI:** 10.3390/s21030740

**Published:** 2021-01-22

**Authors:** Jie Wu, Hongjun Xie, Alireza Sadr, Kwok-Hung Chung

**Affiliations:** 1 Department of Dentistry, Shandong Medical College, 6 Jucai Rd, Linyi 276000, Shandong, China; wujie@sdmc.edu.cn; 2 Department of Oral and Maxillofacial Surgery, Linyi People’s Hospital, 27 Jiefang Road, Linyi 276000, Shandong, China; hongjunxie@outlook.com; 3 Department of Restorative Dentistry, School of Dentistry, Box 357456, University of Washington, Seattle, WA 98195-7456, USA; arsadr@uw.edu

**Keywords:** provisional crowns, internal fitness, marginal adaptation, fit checking method, polyvinyl siloxane-replica method, optical coherence tomography, 3D printing, milling

## Abstract

Different techniques have been used to construct provisional crowns to protect prepared teeth. The purpose of this in vitro study was to assess the internal fit and marginal discrepancy of provisional crowns made by different methods. A total of 48 provisional crowns were constructed and divided into three groups (*n* = 16) according to the fabrication methods: fabricated manually-group MAN; computer-aided design/computer aided manufacturing technology-group CAM; and 3-dimensional (3D)-printed technology-group 3DP. The same standard tessellation language (STL) file was used for both CAD/CAM and 3D-printed group. The silicone-checked method was used to measure the internal gap distance. The marginal discrepancy was measured by using the polyvinyl siloxane (PVS) replica method and swept-source optical coherence tomography (OCT) scanning technique. Data were analyzed with one-way analysis of variance (ANOVA) nonparametric Kruskal-Wallis and Tukey tests at α = 0.05. At the central pit and axial walls, the gap distance mean values of group CAM were higher than those from group MAN and 3DP. The group 3DP was statistically significantly higher in gap distance at the location of occlusion than group MAN and group CAM (*p* < 0.05). The total gap distances assessed by silicone-checked method revealed there were no statistically significant differences between the tested groups (*p* > 0.05). The total mean values of absolute and horizontal marginal discrepancy of the group 3DP obtained by using the PVS-replica method and OCT scanning technique were significantly higher than the group MAN and CAM (*p* < 0.05). Regression correlation results of marginal discrepancy indicated a positive correlation (*r* = 0.902) between PVS-replica method and OCT scanning technique. The manually fabricated provisional crowns presented better internal fit and a smaller marginal discrepancy. Between different assessment techniques for marginal adaptation, PVS-replica method and OCT scanning technique have a positive correlation.

## 1. Introduction

Provisional crowns are essential to protect the vital tissue of prepared teeth and periodontal tissues [1]. They are also used to maintain the oral function and esthetics [1,2]. Internal fit and marginal adaptation are the key criteria for the long-term clinical success of any restorations [3,4,5,6]. Excellent internal fit will facilitate crown seating without compromising retention and resistance forms during cementation [7,8]. Poor marginal fit can enhance microleakage and plaque accumulation, with subsequent cement dissolution, recurrent decay, and periodontal inflammation occurrence [9,10]. Therefore, special care should be taken to ensure the internal fit and marginal adaptation of restorations.

The fit of a provisional crown is closely related to the fabrication method, which could be a direct or indirect process [11,12]. In the direct method, the provisional restoration is fabricated immediately on the prepared teeth, which is straightforward and relatively simple [13]. The most commonly used material in the direct method is polymethyl methacrylate (PMMA) resin [14]. However, the exothermic reaction during polymerization may cause thermal damage to the pulp irreversibly [15]. Moreover, polymerization shrinkage may cause dimensional change especially in the marginal regions causing marginal leakage [16,17]. Recently, bis-acryl composite resin has been used for constructing provisional crowns and is considered to mitigate some shortcomings of PMMA resin material [18].

Computer-aided design and computer-aided manufacturing (CAD/CAM) technology has recently been applied to the fabrication of provisional crowns as an indirect technique [19]. At present, most commercially available dental CAD/CAM systems use the milling or subtractive method to sculpt a resin block or pluck by using cutting instruments into the desired shape [19]. The CAD/CAM technology could reduce laboratory time, increase productivity, and result in high quality products consistently when compared to the conventional direct technique [20]. However, CAD/CAM technology may result in the waste of raw materials and need of strict maintenance. In addition, because of some limitations of the motion range of the cutting device, CAD/CAM restorations may have poor micro reproducibility especially in the curved surfaces or regions [21,22].

In the recent dental restoration processing field, to avoid the shortcomings of the CAD/CAM milling system (subtractive manufacturing), the three-dimensional (3D) printing system (additive manufacturing) has been introduced, which is believed to have the strength to manufacture the precise prosthesis with less materials [19,23]. The 3D printing technology builds up the product in a layer-to-layer fashion from CAD data. Each layer is polymerized and combined with the previous layer by photopolymerization at once [24]. However, significant polymerization shrinkage has been reported, which is caused by the reduction of the atomic distance in the low-molecular-weight monomers during the material-layering and polymerization [21].

Cement space thickness is the crucial element for the accuracy of restoration margins and has a significant impact on the flexural failure load of restorations [25,26]. Thus, several evaluation methods were introduced to assess the internal and marginal fit, such as dye and tracer penetration tests, silicone replica technique, and microcomputed tomography (micro-CT) [27,28]. Optical coherence tomography (OCT) is a nondestructive method that utilizes a beam of partially coherent light to reconstruct the tomographic images. OCT can provide better image resolution and scanning speed in real-time and in contactless mode, using non-ionizing radiation, unlike magnetic resonance imaging and ultrasounds. Recent studies showed OCT has been used in the detection of enamel cracks, dental caries, and periodontal problems [29,30,31,32].

The aim of this study is to evaluate the internal fit and marginal discrepancy of the provisional crowns made with manually fabricated technique, CAD/CAM milling technology, and 3D printing technology. The null hypothesis was that there would be no differences in the fit of the provisional crowns manufactured by using the above manufacturing techniques.

## 2. Materials and Methods

### 2.1. Specimen Preparation

A mandibular left first molar (#36) resin tooth (Columbia Dentoform Corp., Lancaster, PA, USA) was scanned using a 3D laboratory scanner (D2000, 3Shape, Copenhagen, Denmark) and saved in a standard tessellation language (STL) file format before crown preparation. The resin tooth was then prepared with a fine diamond bur (no. 6856, Brasseler USA, Savannah, GA, USA) as follows: 2.0 mm occlusal reduction, and 1.0 mm axial wall contouring with a 1.0 mm rounded shoulder finish line circumferentially, and 12 degrees total occlusal convergence manufactured by a customized grinding jig. The maxillary and mandibular models (Columbia Dentoform, Long Island City, NY, USA), prepared resin tooth #36, and the occlusal relationship were scanned using a model scanner (D2000, 3Shape) to obtain the data for milling the maxillary and mandibular model as well as printing the removable #36 resin dies. The data of model scanning were manipulated in a CAM software program to mill the maxillary and mandibular models with composite resin material (EB700 High Temperature Epoxy Tooling Board, Easy Composite Ltd., Staffordshire, UK) by using a CAM milling machine (TDS ME300; Pouyu Biotech, TAIWAN) according to the manufacturer’s specifications [1]. The scanned data were manipulated with a CAD software and connected to a 3D printer (Ededn500V, Stratasys Direct, Inc., Valencia CA, USA) to fabricate stereolithographic resin dies (MED690, Stratasys Direct Manufacturing, Valencia, CA, USA). Based on the data from a pilot study, the sample size (*n* = 16 per group) was estimated with a power analysis to provide statistical significance (α = 0.05 at 85%). Forty-eight 3D-printed resin dies were constructed accordingly and equally divided into three groups and three different provisional crowns were fabricated by using different procedures. Materials used in this study are summarized in Table 1.

For the manual group (Group MAN), a conventional direct technique was used to fabricate the provisional crowns. The auto-polymerized and resin-based composite material (LuxaCrown, DMG America, Ridgefield Park, NJ, USA) was loaded into a silicone matrix before crown preparation made with PVS material (Cinch, Parkell Inc., Edgewood, NY, USA) using an automix-dispenser, and then placed on the #36 resin die and left for 3 min until the initial set of the composite material. Sixteen provisional crowns were fabricated directly according to the above protocol. The provisional crowns were then finished and polished accordingly under a 10× magnification dental laboratory microscope (Vision, LW Scientific, Lawrenceville, GA, USA).

For the CAD/CAM group (Group CAM), the STL file created before crown preparation and the STL file data for printing resin dies aforementioned were used to design the milling provisional crowns using CAD software (3Shape). Sixteen CAD-CAM provisional crowns with 60 µm die spacer, were milled by using a 5-axis dental milling machine (DWX-51D, Roland DGA, Frenchs Forest, NSW, Australia) from the resin nano ceramic material (Lava Ultimate, 3M ESPE, St. Paul, MN, USA) blocks.

For the 3D printing group (Group 3DP), the STL file of unprepared resin tooth and the STL file data of prepared #36 resin tooth for printing dies were imported to the CAD software described previously. Sixteen 3D-printed provisional crowns were constructed with printed methacrylate material (Dima Denture teeth, Kulzer North America, South Bend, IN, USA) and 3D printer (cara^®^ Print 4.0, Kulzer North America, South Bend, IN, USA) using 3D printing technology according to manufacturer’s instructions.

### 2.2. Measurement of Internal Fit

**Figure 1:**
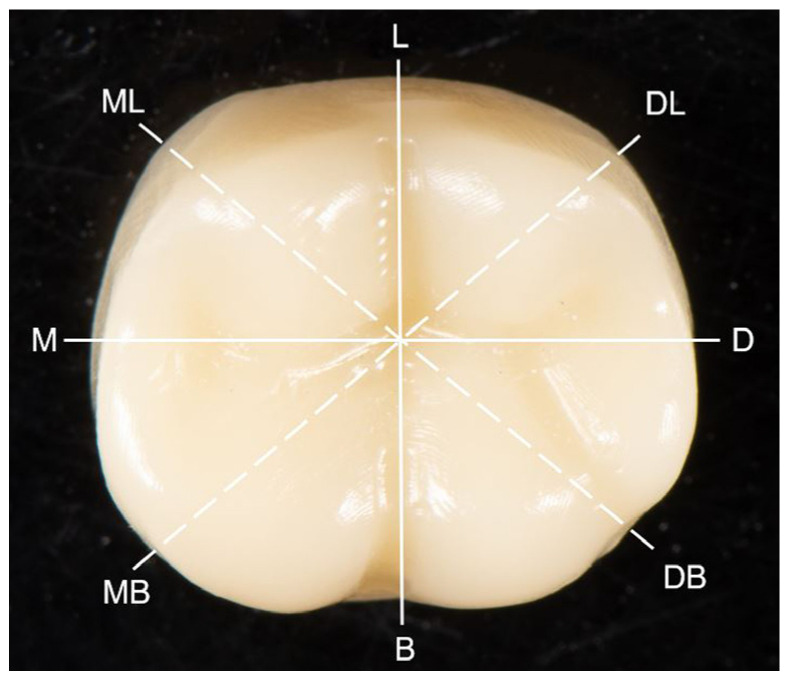
Proposed section orientation of silicone-checked materials. Lines indicate the cutting direction; B: buccal, D: distal, L: lingual, M: mesial, DB: distal and buccal, DL: distal and lingual, ML: mesial and lingual, MB: mesial and buccal.

**Figure 2:**
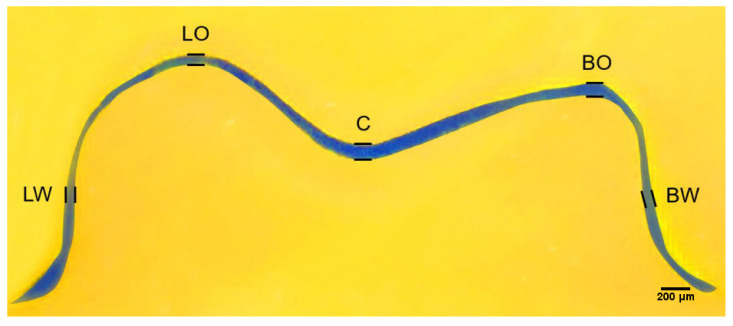
Representative cross-sectional view through central pit in mesial-distal direction. BW: midpoint at the buccal axial wall; BO: buccal-occlusal gap; C: central pit; LW: midpoint at the lingual axial wall; LO: lingual-occlusal gap.

Each provisional crown was luted on a 3D-printed die with a fit checking silicone material (Fit Checker Advanced Blue, GC, Tokyo, Japan) under a 50 N load for 10 min to simulate the clinical luting procedure. Following the removal of the excess material at the marginal areas, each provisional crown-die pair was weighed before and after luting. After subtracting the weight of crown-die pair before luting with the silicone material, the volume of silicone material inside each crown was calculated from its weight and density. After 10 min bench polymerization of the silicone materials, provisional crowns were removed from the dies carefully, and the entire crown portion of the resin die surface was covered with light body PVS material (Aquasil Ultra XLV; Dentsply Sirona, Charlotte, NC, USA) to envelope the retained fit checking silicone material. Then, the dies were gently separated from the silicone materials, and the light body PVS material (Aquasil Ultra XLV) was again injected onto the intaglio surface to form a sandwich-like silicone block for the purpose of embedding the blue silicone film that represented the gap space or cement space between the provisional crown and resin die. To prepare cross-sectioned specimens for internal gap assessment, all the silicone blocks were labeled and cut with a no. 11 surgical scalpel blade (Henry Schien Inc., Melville, NY, USA) in the buccal to lingual, mesial to distal, mesiobuccal to distolingual, and mesiolingual to distobuccal directions (Figure 1) [1]. Using this silicone-checked method, an approximate 1.0 mm thick silicone material was sectioned along the above directions. Eight points at the middle of the axial walls and occlusal peaks as well as the central pit correlated with the above sectional directions were defined (Figure 2). The thickness or width of the blue silicone material at the above 17 locations were then measured accordingly using a measuring microscope (FMA050; AmScope, Irvine, CA, USA) at 10× magnification. Data were used to present the internal fit of the provisional crowns.

### 2.3. Measurement of Marginal Discrepancy

Following the internal fit checking procedure, the provisional crowns and resin dies were cleansed thoroughly by using steam pressure (Aquaclean 3; Degussa Dental, Wolfgang, Germany) and then ultrasonic cleaner with distilled water for 5 min, and bench dried. The provisional crown was then cemented on its corresponding resin die by using Temp-Bond NE (Kerr Corp., Orange, CA, USA) according to the manufacturer’s instructions under a 50 N load for 10 min. After excess cement removal and water bath immersing at 37 °C for 24 h, the marginal adaptation of the provisional crowns was evaluated by using the PVS-replica method and swept source optical coherence tomography (SS-OCT) scanning technique, respectively.

#### 2.3.1. PVS-Replica Method

For assessment with PVS-replica method, light-body PVS impression material (Aquasil Ultra XLV) was placed at the margin of each cemented provisional crown extending 2 mm above and below the cemented margin for a setting time of 6 min on bench. The marginal discrepancy was measured at eight selected locations along the margin circumferentially including buccal (B) distal (D), lingual (L), and mesial (M), and the midpoint of buccal and distal (BD), distal and lingual (DL), lingual and mesial (LM), mesial and buccal (MB) as described in Figure 1. The PVS impression material was cut perpendicularly along the margin with a no. 11 surgical scalpel blade (Henry Schein, Inc., Melbill, NY, USA) at the selected locations to section a marginal replica specimen with 0.5 mm in thickness. The sectioned specimens were examined and measured by using a stereoscope (Measurescope 20; Nikon, Tokyo, Japan) at 75× magnification. Horizontal and vertical marginal discrepancies were measured, and the absolute marginal discrepancy (mm) was calculated accordingly at each point based on the Holmes’s recommendation [33].

#### 2.3.2. OCT Scanning Technique

For measurement with OCT scanning, the SS-OCT system (Yoshida Dental OCT, Yoshida Dental Mfg, Tokyo, Japan) was used in this study to construct 3D images with a central wavelength of 1310 nm and a scan range of 140 nm at 50 KHz. The optical resolution of the 3D dataset in air was 11 μm in depth and 40 μm in lateral and axial dimensions. Each crown-die assembly was scanned at a fixed distance to take 3D images at eight sites as described previously for the PVS-replica method, which were B, D, L, M, BD, DL, LM, and MB, respectively (Figure 1). The horizontal and vertical marginal discrepancies at each point was measured on scan slices obtained from the 3D images in pixels using ImageJ software and then converted to micrometer unit.

### 2.4. Statistical Analysis

The internal fit and marginal discrepancy values of the provisional crown are presented as the means and standard deviations. Comparisons were performed with the one-way ANOVA analysis and Tukey honestly significance difference (HSD) tests for the data from cement space volume and internal fit assessment. Nonparametric Kruskal–Wallis test was used for marginal discrepancy data analysis. The correlations between the measurements of PVS-replica method and OCT scanning technique were assessed using Pearson’s correlation coefficient. SPSS for Windows (version 20, SPSS Inc., Chicago, IL, USA) was used for all statistical analyses. The statistical significance was determined based on the significant level of 0.05.

## 3. Results

**Figure 3:**
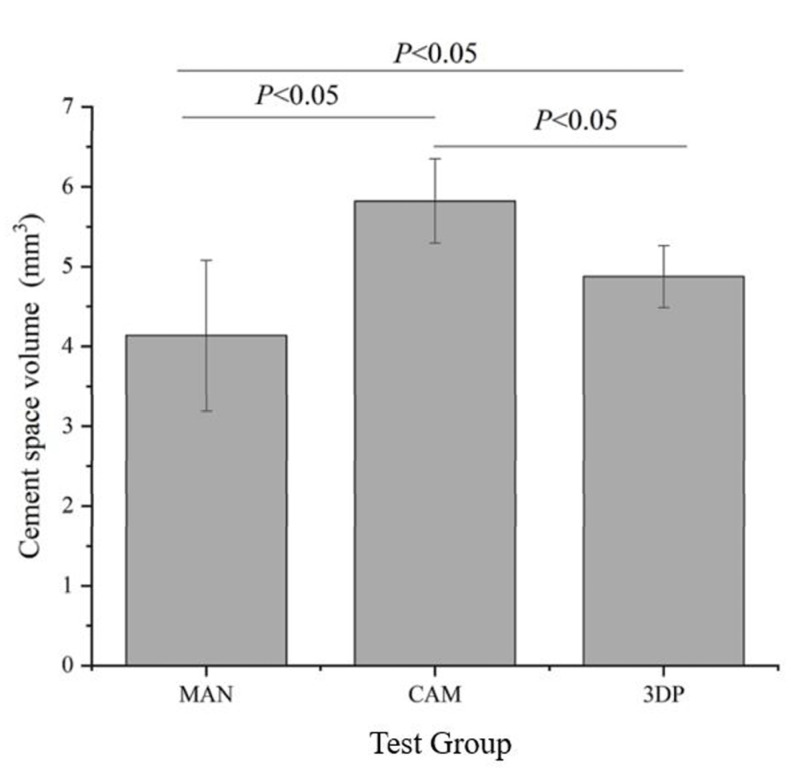
Results of cement space volume measurements from silicone-checked method.

**Figure 4:**
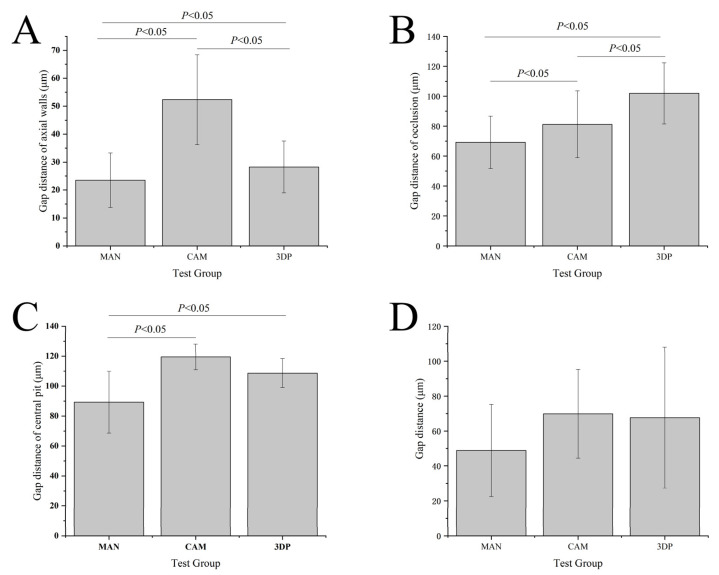
The results of the gap distances of test crowns measured from polyvinyl siloxane-replica method in (**A**) gap distance of axial walls, (**B**) gap distance of occlusion, (**C**) gap distance of central pit, and (**D**) total gap distance. MAN: manual group, CAM: CAD/CAM group, and 3DP: 3D printed group. The columns connected by horizontal bars were significantly different.

The cement space volume values of the group CAM (5.8 ± 0.5 mm^3^) were statistically significant (F = 25.93) higher than the group MAN (4.1 ± 0.9 mm^3^, *p* < 0.05) and group 3DP (4.9 ± 0.4 mm^3^, *p* < 0.05), respectively, are shown in Figure 3. The results of internal fit assessment by silicone-checked method are shown in Figure 4A–D. The gap distance mean of group CAM at the axial walls (52.3 ± 16.1 μm) were statistically significantly (F = 208.39) higher than group MAN (23.5 ± 9.7 μm, *p* < 0.05) and group 3DP (28.3 ± 9.3 μm, *p* < 0.05), respectively. The group 3DP (101.9 ± 20.4 μm) was statistically significantly (F = 86.10) higher in gap distance at the location of occlusion than group MAN (69.2 ± 17.4 μm, *p* < 0.05) and group CAM (81.3 ± 22.4 μm, *p* < 0.05), respectively. At the location of central pit, the gap distance mean values of group CAM (119.5 ± 8.6 μm) were statistically significantly (F = 18.99) higher than those from group MAN (89.3 ± 20.6 μm) and group 3DP (108.7 ± 9.7 μm), but there was no statistical difference between group CAM and group 3DP (*p* > 0.05). Furthermore, there were no statistically significant differences in the total gap distance of the three types of provisional crowns in the internal fit assessing by using silicone-checked method (*p* > 0.05).

**Figure 5:**
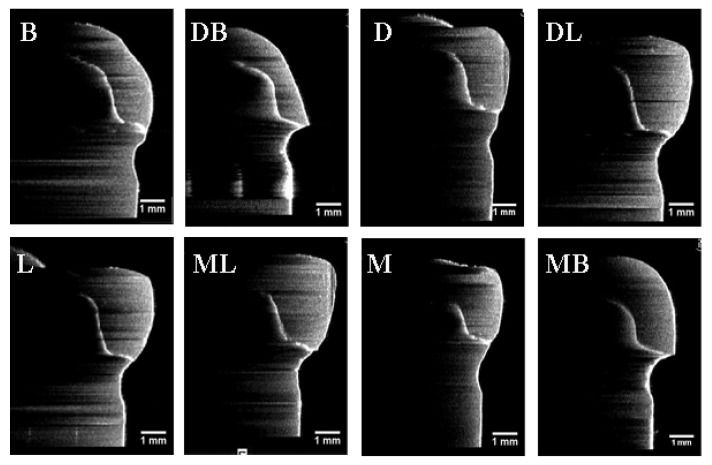
Images of the optical coherence tomographic scans of a representative manual provisional crown (Group MAN) at eight selected locations. (B) At buccal point, (DB) at distal-buccal point, (D) at distal point, (DL) at distal-lingual point, (L) at lingual point, (ML) at mesial-lingual point, (M) at mesial point, and (MB) at mesial-buccal point. All the orientations of the OCT images have been rotated by 90 degrees from the original orientation of the OCT images.

**Figure 6:**
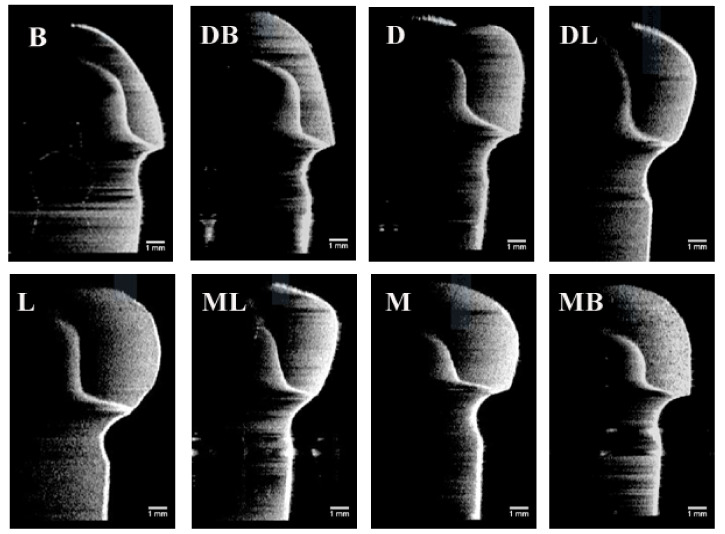
Images of the optical coherence tomographic scans of a representative CAD/CAM provisional crown (Group CAM) at eight selected locations. (B) At buccal point, (DB) at distal-buccal point, (D) at distal point, (DL) at distal-lingual point, (L) at lingual point, (ML) at mesial-lingual point, (M) at mesial point, and (MB) at mesial-buccal point. All the orientations of the OCT images have been rotated by 90 degrees from the original orientation of the OCT images.

**Figure 7:**
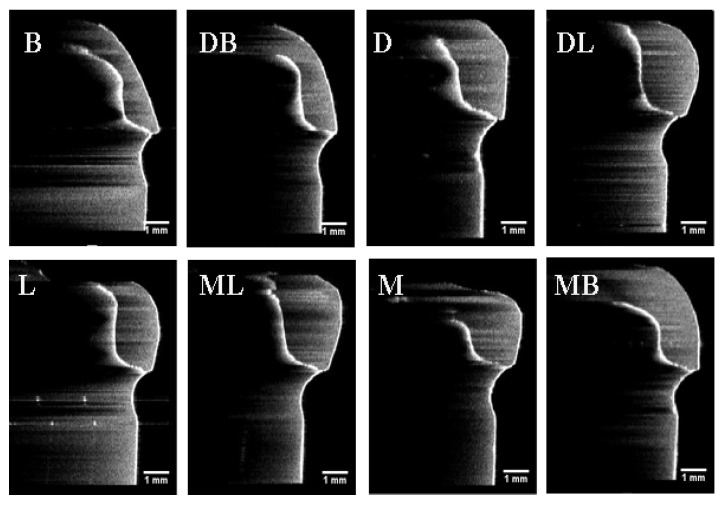
Images of the optical coherence tomographic scans of a representative 3D printed provisional crown (Group 3DP) at eight selected locations. (B) At buccal point, (DB) at distal-buccal point, (D) at distal point, (DL) at distal-lingual point, (L) at lingual point, (ML) at mesial-lingual point, (M) at mesial point, and (MB) at mesial-buccal point. All the orientations of the OCT images have been rotated by 90 degrees from the original orientation of the OCT images.

**Figure 8:**
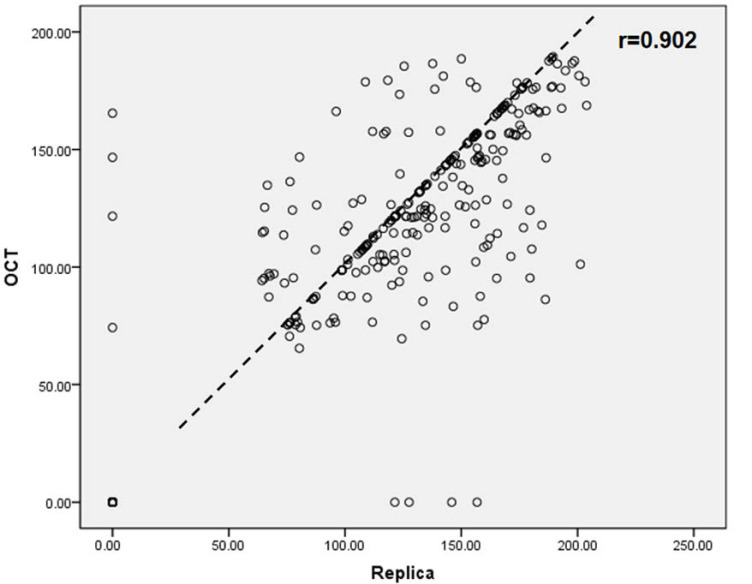
Regression correlation results of marginal discrepancy obtained by polyvinyl siloxane-replica method and optical coherence tomographic scanning technique.

The results of absolute marginal discrepancy, horizontal, and vertical marginal discrepancy mean values are measured and calculated. The results are listed in Table 2, Table 3 and Table 4. For the absolute marginal discrepancy at the selected eight sites (Table 2), the means and standard deviations from 3DP group higher than that of CAM and MAN group (*p* < 0.05), except the means of mesial site by PVS method. The 3DP provisional crowns obtained the total mean values of absolute marginal discrepancy measurements, that are statistically significantly higher than the group MAN and CAM, respectively, (*p* < 0.05), but there was no statistical difference between group CAM and group MAN (*p* > 0.05). The total mean values of vertical marginal discrepancy obtained from each group are relatively low, and ranged from 7.0 μm to 21.5 μm measured by the PVS-replica method and 4.5 μm to 20.3 μm by OCT scanning technique, as listed in Table 4. Representative provisional crown OCT scan images from each group at eight selected locations using OCT scanning technique are presented in Figure 5, Figure 6 and Figure 7. The Pearson correlation test determined that there is a positive correlation (*r* = 0.902) between the PVS-replica method and OCT scanning technique (Figure 8).

## 4. Discussion

This current study was to compare the internal fit and marginal discrepancy of three groups of provisional crowns fabricated by manual technique, CAD/CAM milling method, and 3D printed technology, respectively. Internal fit was assessed indirectly using the silicone-checked method to calculate the cement space volume and PVS-replica method to measure the marginal discrepancy which is known as the nondestructive and accurate techniques [34,35]. Results indicate that there are statistically significant differences in the calculated cement space volumes and marginal discrepancy between the tested groups. However, the internal fit assessment before final cementation using PVS-replica method showed no significant difference in total gap distances between three types of provisional crowns as shown in Figure 4D. The total marginal discrepancy means of group MAN were lower than total mean values from group CAM and 3DP. This might be attributed to the preset of the virtual crowns of CAD/CAM and 3DP systems with 60 μm cementation space. At the location of central pit, the gap distance total mean values of group CAM were higher than those from group MAN and 3DP (Figure 4C). In addition, the internal gap distance total mean values at the area of axial walls with curved surface also showed higher values in the provisional crowns made by CAD/CAM technology (Figure 4). Because of the limited size and angle of the cutting tools, CAD/CAM technology usually may have some limitations to processing the intaglio surface of prostheses correspondingly [21,22]. The results of the present study were consistent with Lee et al. [36] and Mai et al. [12] published literatures that reported that the relatively uneven surfaces or regions would be more difficult to reproduce perfectly in the CAD/CAM technology. Therefore, the null hypotheses of no significant differences in the internal fit and marginal discrepancy between provisional crowns fabricated by different manufacturing methods were partially rejected.

Marginal adaptation is crucial to the clinical success of dental restorations [9,10]. In general, the accuracy of marginal fit depends on teeth preparation, impression technique, materials and technology used to fabricate the restoration and even on the luting cement. Based on published data, McLean et al. [37] considered 120 µm as the maximum margin gap to be clinically accepted, and Boening et al. [38] stated the marginal gap between 100 µm and 200 µm might exist within the clinically permitted range. In current study, the 3DP group showed the highest absolute marginal discrepancy obtained by the PVS-replica method (120.8 ± 70.9 µm) and OCT scanning technique (143.1 ± 39.9 µm), which is within clinically accepted range mentioned previously (Table 2). It was found that the total mean values of marginal gap recorded for the MAN group were 71.3 ± 64.9 µm by PVS-replica method and 82.7 ± 65.8 µm by OCT scanning technique, which were less than those from the 3DP and CAM groups. These findings are different from some previous investigations, which reported that the 3D printed restorations exhibit significantly lower marginal gap values than their manual and milled counterparts [12,34]. In fact, many factors, and parameters, such as different printers, shrinkage between layers, polymerizing temperature, and minimal thickness of the layer, might attribute to the different results [39,40]. Tahayeri et al. [41] characterized the stereolithography 3D printing parameters relevant to optimize and achieve consistent and accurate printing results and concluded that the accuracy of a 3D printed provisional crown varied considerably depending on the orientation of the printed part and the area of the structure accuracy was measured. The closer the supports to the marginal region of the provisional crown would be more difficult to conduct finishing and polishing the prostheses precisely after printing. Furthermore, in this study, for the MAN group, bis-acryl composite resin was chosen instead of conventional PMMA, which was reported to have superior clinical performance and esthetic longevity than conventional PMMA [18]. In the present study, the absolute marginal discrepancy was calculated by horizontal and vertical discrepancy based on the Holmes’s design [33]. It was found that the horizontal marginal gap mean values recorded for the three groups were higher than that of vertical marginal discrepancy in Table 3 and Table 4. Previous studies have reported that overextended margins exert more negative influence on the periodontal health, such as excessive plaque retention, gingival inflammation, and even rapid alveolar bone destruction [42,43].

Nawafleh et al. [44] indicated that an ideal evaluation of marginal discrepancy is hardly obtained using single technique. In the present study, both the PVS-replica method and OCT scanning technique were used to assess the marginal discrepancy, which are known as noninvasive and effective techniques. However, it is reported that the accuracy of PVS-replica method may be affected by the type of silicone materials, the precision of the measuring procedure, and the number of the measuring points [45]. OCT scanning technique is a non-invasive method with a 2–3 mm penetration depth, high resolution, and real-time view of the image, which have been applied for oral screening and diagnosis of early detection of caries, oral mucosa, periodontal tissues, and melanoma [29,30,31,46]. Micro-CT imaging is a diagnostic transmission imaging technique that utilizes ionizing radiation, which has been used to evaluate the fit and marginal adaptation of interim crowns [32]. Compared to OCT, micro-CT is a relatively expensive method; and it produces a high-dose radiation when testing the samples in contactless mode [47]. Furthermore, micro-CT requires the rotation of samples to reconstruct 3D models; however, OCT can provide real-time 2D images and 3D scans without the need for sample rotation. Therefore, OCT is considered to detect interfacial gaps more accurately [32].

The images are obtained by using the OCT scanning of representative manual, CAD/CAM milled, and 3D-printed provisional crowns at eight measurement points, which are intuitive and easy to measure the marginal discrepancy. According to the results of Table 2, Table 3 and Table 4, the mean values of marginal discrepancy of the three groups measured by the silicone replica technique and OCT have similar analysis results. Moreover, the regression analysis revealed that a positive coefficient of determination (R^2^ = 0.902) was found between the data obtained from PVS-replica method and OCT scanning technique, which suggested the consistency of these two types of measurements. As a noninvasive and accurate method, OCT has great potential to become a clinical testing tool in routine dental practice.

About conducting the tests with commercial provisional materials, one of the limitations of this design was its lack of unified composition of the test resin-based materials. A universal provisional crown material should be formulated in studying different manufacturing methods, which forms the basis for other future studies in our laboratory.

## 5. Conclusions

Within the limitations of the current in vitro study, the different manufacturing methods influence the internal fit and the marginal discrepancy of provisional crowns. The manually fabricated provisional crowns using resin-based composite material presented lower gap distance which means better internal fit and a smaller absolute marginal discrepancy. Between different assessment techniques for marginal adaptation, PVS-replica method and OCT scanning technique have a positive correlation.

## Figures and Tables

**Table 1:** List of materials used in this study.

Material	Code	Fabricating Method	Manufacturer	Batch no.
LuxaCrown	MAN	Manual	DMG, Hamburg, Germany	788811
Lava Ultimate	CAM	CAD/CAM Technology	3M ESPE, St. Paul, MN, USA	2914B1-LT/14L
Dima Print Denture Teeth	3DP	CAD/3D Printing	Kulzer North America South Bend, IN, USA	AC18124A2
Fit Checker Advanced Blue		Automix	GC Corp., Tokyo, Japan	1512021
Aquasil Ultra XLV		Automix	Dentsply Sirona, Charlotte, NC, USA	170524

**Table 2:** Results of absolute marginal discrepancy (μm) using polyvinyl siloxane-replica method and optical coherence tomography technique.

Marginal Discrepancy (μm)	B Mean ± SD	DB Mean ± SD	D Mean ± SD	DL Mean ± SD	L Mean ± SD	ML Mean ± SD	M Mean ± SD	MB Mean ± SD	Total Mean ± SD
**Polyvinyl siloxane-replica method**									
**MAN**	91.3 ± 64.1	65.6 ± 65.9	59.9 ± 50.2	90.0 ± 76.5	84.3 ± 70.8	38.9 ± 54.7	48.8 ± 54.9	91.7 ± 67.7	71.3 ± 64.9 ^a,b^
**CAM**	127.4 ± 33.9	134.7 ± 46.4	120.8 ± 44.2	69.7 ± 57.3	64.1 ± 76.3	65.9 ± 56.6	93.3 ± 60.9	109.8 ± 53.0	96.9 ± 60.2 ^b^
**3DP**	129.9 ± 68.4	143.1 ± 45.6	151.5 ± 49.4	129.3 ± 78.5	123.1 ± 75.2	95.6 ± 80.3	78.4 ± 74.7	115.9 ± 71. 7	120.8 ± 70.9 ^c^
**Optical coherence tomographic scanning technique**									
**MAN**	110.4 ± 58.9	73.7 ± 67.9	97.0 ± 59.2	98.3 ± 71.1	52.8 ± 55.7	48.6 ± 65.7	77.8 ± 66.1	102.8 ± 64.7	82.7 ± 65.8 ^a,b^
**CAM**	111.6 ± 28.7	144.5 ± 30.2	130.4 ± 30.4	83.6 ± 70.5	30.3 ± 56.7	91.8 ± 44.5	100.0 ± 53.0	104.9 ± 28.9	99.6 ± 54.6 ^b^
**3DP**	145.5 ± 37.4	147.3 ± 32.2	149.1 ± 44.1	158.0 ± 35.7	127.4 ± 47.5	148.2 ± 29.6	137.2 ± 47.5	132.4 ± 40.1	143.1 ± 39.9 ^c^

B: at buccal point; DB: distal-buccal point; D: distal point; DL: distal-lingual point; L: lingual point; ML: mesial-lingual point; M: mesial point; MB: mesial-buccal point. Different superscript letters indicate significant differences between groups (*p <* 0.05).

**Table 3:** Results of horizontal marginal discrepancy (μm) using polyvinyl siloxane-replica method and optical coherence tomography technique.

Marginal Discrepancy (µm)	B Mean ± SD	DB Mean ± SD	D Mean ± SD	DL Mean ± SD	L Mean ± SD	ML Mean ± SD	M Mean ± SD	MB Mean ± SD	Total Mean ± SD
**Polyvinyl siloxane-replica method**									
**MAN**	83.7 ± 55.5	62.2 ± 58.6	55.7 ± 48.0	68.6 ± 55.7	79.7 ± 67.1	33.0 ± 44.4	54.4 ± 52.5	69.00 ± 52.7	63.3 ± 55.2 ^a^
**CAM**	118.5 ± 27.9	136.4 ± 42.5	128.3 ± 42.4	69.7 ± 57.3	56.8 ± 76.6	59.2 ± 54.9	94.5 ± 61.0	95.1 ± 53.1	94.8 ± 59.8 ^b^
**3DP**	116.1 ± 72. 5	139.3 ± 45.1	148.8 ± 47.7	127.9 ± 64.7	116.9 ± 72.6	94.5 ± 69.7	86.6 ± 81.6	117.7 ± 72.3	118.7 ± 67.7 ^c^
**Optical coherence tomographic scanning technique**									
**MAN**	93.3 ± 54.2	65.7 ± 61.9	94.0 ± 51.5	82.6 ± 61.1	57.1 ± 64.1	45.5 ± 61.2	67.0 ± 66.2	76.1 ± 49.2	72.7 ± 59.5 ^a^
**CAM**	115.6 ± 28.0	132.3 ± 36.2	128.5 ± 30.4	82.3 ± 69.8	33.8 ± 63.8	71.7 ± 44.1	101.6 ± 46.6	110.1 ± 27.6	97.0 ± 54.3 ^b^
**3DP**	152.4 ± 27.3	140.6 ± 28.0	149.8 ± 40.7	152.7 ± 24.0	145.7 ± 45.5	150.0 ± 29.9	139.1 ± 50.0	140.6 ± 43.2	146.4 ± 36.6 ^c^

B: at buccal point; DB: distal-buccal point; D: distal point; DL: distal-lingual point; L: lingual point; ML: mesial-lingual point; M: mesial point; MB: mesial-buccal point. Different superscript letters indicate significant differences between groups (*p <* 0.05).

**Table 4:** Results of vertical marginal discrepancy (μm) using polyvinyl siloxane-replica method and optical coherence tomography technique.

Marginal Discrepancy (µm)	B Mean ± SD	DB Mean ± SD	D Mean ± SD	DL Mean ± SD	L Mean ± SD	ML Mean ± SD	M Mean ± SD	MB Mean ± SD	Total Mean ± SD
**Polyvinyl siloxane-replica method**									
**MAN**	40.7 ± 45.3	28.5 ± 33.0	14.8 ± 21.5	18.2 ± 26.0	15.1 ± 24.5	10.5 ± 18.7	14.4 ± 23.2	21.9 ± 32.0	21.5 ± 30.3 ^a^
**CAM**	6.3 ± 17.9	19.1 ± 32.9	3.7 ± 14.3	0	2.3 ± 8.8	0	7.2 ± 19.9	18.7 ± 32.2	7.0 ± 20.1 ^b^
**3DP**	25.0 ± 26.5	8.3 ± 17.9	11.0 ± 24.0	11.8 ± 25.5	9.7 ± 21.1	11.1 ± 24.3	5.3 ± 14.4	23.8 ± 33.2	13.3 ± 24.2 ^b,c^
**Optical coherence tomographic scanning technique**									
**MAN**	41.4 ± 52.4	25.5 ± 42.3	22.4 ± 47.0	8.4 ± 26.1	3.6 ± 14.5	0	21.5 ± 46.4	39.7 ± 55.4	20.3 ± 41.5 ^a^
**CAM**	2.8 ± 11.3	18.5 ± 28.8	5.7 ± 15.5	0	2.8 ± 11.1	2.8 ± 11.1	0	2.9 ± 11.4	4.5 ± 14.8 ^b^
**3DP**	18.8 ± 25.2	6.7 ± 18.4	3.6 ± 14.5	11.5 ± 25.2	6.4 ± 17.6	3.5 ± 14.2	0	4.2 ± 16.6	6.8 ± 18.4 ^b,c^

B: at buccal point; DB: distal-buccal point; D: distal point; DL: distal-lingual point; L: lingual point; ML: mesial-lingual point; M: mesial point; MB: mesial-buccal point. Different superscript letters indicate significant differences between groups (*p* < 0.05).
